# Sex Disparities in the Association of Serum Uric Acid With Kidney Stone: A Cross-Sectional Study in China

**DOI:** 10.3389/fmed.2022.774351

**Published:** 2022-02-09

**Authors:** Jin-Zhou Xu, Jun-Lin Lu, Liu Hu, Yang Xun, Zheng-Ce Wan, Qi-Dong Xia, Xiao-Yuan Qian, Yuan-Yuan Yang, Sen-Yuan Hong, Yong-Man Lv, Shao-Gang Wang, Xiao-Mei Lei, Wei Guan, Cong Li

**Affiliations:** ^1^Department of Urology, Tongji Medical College, Tongji Hospital, Huazhong University of Science and Technology, Wuhan, China; ^2^Department of Urology, Sun Yat-sen Memorial Hospital, Sun Yat-sen University, Guangzhou, China; ^3^Health Management Center, Tongji Medical College, Tongji Hospital, Huazhong University of Science and Technology, Wuhan, China

**Keywords:** kidney stone, uric acid, sex disparities, malnutrition, cross-sectional study, restricted cubic splines

## Abstract

**Background and Aims:**

Urolithiasis is characterized by high rates of prevalence and recurrence. Hyperuricemia is related to various diseases. We hope to determine the association between serum uric acid (UA) level and kidney stone (KS).

**Methods:**

In this population-based cross-sectional study, a total of 82,017 Chinese individuals who underwent a comprehensive examination in 2017 were included. The KS was diagnosed based on ultrasonography examination outcomes. Fully adjusted odds ratio (*OR*) for KS, and mean difference between the two groups were applied to determine the association of UA level with KS.

**Results:**

Among the 82,017 participants included in this study (aged 18~99 years), 9,435 participants (11.5%) are diagnosed with KS. A proportion of 56.3% of individuals is male. The mean UA level of overall participants is 341.77 μmol/L. The participants with KS report higher UA level than the participants without KS [mean UA level 369.91 vs. 338.11 μmol/L; mean difference (MD), 31.96 (95% *CI*, 29.61~34.28) μmol/L]. In men, the OR for KS significantly increases from 330 μmol/L UA level. Every 50 μmol/L elevation of UA level increases the risk of KS formation by about 10.7% above the UA level of 330 μmol/L in men. The subgroup analysis for male is consistent with the overall result except for the participants presenting underweight [adjusted *OR*, 1.035 (0.875~1.217); MD, −5.57 (−16.45~11.37)], low cholesterol [adjusted *OR*, 1.088 (0.938~1.261); MD, 8.18 (−7.93~24.68)] or high estimated glomerular filtration rate (eGFR) [adjusted *OR*, 1.044 (0.983~1.108); MD, 5.61 (−1.84~13.36)]. However, no significant association is observed in women between UA and KS either in all female participants or in female subgroups.

**Conclusion:**

Among Chinese adults, UA level is associated with KS in a dose-response manner in men but not in women. However, the association becomes considerably weak in male participants with malnutrition status.

## Introduction

Kidney stone (KS) is one of the most common disorders in the urinary system, with prevalence rates ranging from 1~5% in Asia, 5~9% in Europe, and 7~13% in North America. About 14% of patients will recur within 1 year, while the rate climbs to 25% within 5 years, and 52% within 10 years (1, 2). Owing to the high rates of prevalence and recurrence, KS impairs both the physical and mental health of the patients and causes a heavy financial burden (3). The treatment for KS primarily depends on surgical methods, such as shockwave lithotripsy, ureteroscopy, and percutaneous nephrolithotomy. A patient with KS patient often needs repeated operations that are accompanied by potential complications, such as hemorrhage, urinary tract infection, and ureter stricture (4). Some patients are reported to develop septic shock, loss of kidneys due to hemorrhage, and death. A non-invasive, effective, and persistent therapy is required to prevent the KS formation and relapse (5, 6). It urges a better understanding of the pathophysiological mechanisms during KS development.

Uric acid (UA) is the end product of purine metabolism and excreted by urine. A high level of UA can lower urine pH, leading to inadequate buffer capacity for ammonium salts, eventually resulting in the crystal formation. UA can promote calcium oxide stone through increased urinary excretion of calcium, reduced excretion of citrate, and supersaturation of urine concerning monosodium urate (7). Hyperuricemia was defined as the UA ≥408 μmol/L for male subjects, and ≥360 μmol/L for female subjects (8). However, recent studies reported that the cutoff value might inaccurately predict the morbidity of several diseases, such as KS disease, hypertension, and fatal myocardial infarction (9–12). Kim and colleagues indicated that the risk of KS has a positive correlation with UA level when the UA level is >297 μmol/L (5.0 mg/dl) in the Korean population (9). For KS, no common consensus has been achieved so far for the specific UA level where clinicians can adopt preventive measures.

To obtain a more comprehensive understanding of the role of UA in KS formation in China, we analyze the relationship between serum UA level and KS first in the Chinese population. The research focuses on sex disparities and applies restricted cubic splines to unravel the risk of UA level for KS presence in men and women, respectively. The sex-specific cutoff value of UA level is computed for precise prevention for KS.

## Materials and Methods

### Study Design and Participants

This study was from the project Influencing Factors for Common Chronic Diseases among Chinese Population (IFCCDCP), where individuals underwent a comprehensive test at the Health Management Center of Tongji Hospital from January 1, 2017 to December 31, 2017 (13). This study was approved by the institutional review board of Tongji Hospital, Tongji Medical College, Huazhong University of Science and Technology (Approval ID: TJ-C20160115). The study conformed to the ethical guidelines of the Declaration of Helsinki. Written informed consent was obtained from each participant.

The information and test results of total 99,859 individuals were collected. Participants were excluded if they were under 18 years old (*n* = 351), or they had kidney deformity (*n* = 14), kidney transplantation (*n* = 23), solitary kidney (*n* = 205), or the ultrasonography outcome was absent (*n* = 1,267). Since the data were missing in some participants in a pairwise pattern (missing not at random), we used the deletion method to process the missing data (*n* = 16,492). Finally, a total of 82,017 participants were included in the formal analysis.

### Outcome and Covariates Acquisition

The primary outcome was the presence of KS. KS was confirmed based on an ultrasonography (US) examination. Experienced radiologists without acknowledgment of the study performed a routine abdominal US examination on the participants. Structures that were reported as a strong echo in the renal sinus with posterior acoustic shadows or comet tail signs were regarded as KS.

The selection of covariates was mainly based on a comprehensive literature review for the risk of KS. Demographic characteristics and medical history of hypertension (blood pressure ≥140/90 mmHg), diabetes (fasting blood glucose ≥7.0 mmol/L, or 2-h postprandial blood glucose ≥11.1 mmol/L), and coronary heart disease (CHD) were collected based on the medical history. Physical characteristics, such as body mass index (BMI, calculated as weight in kilograms divided by height in meters squared) and blood pressure were measured by trained nurses. Blood parameters, such as fasting glucose (Glu), alanine aminotransferase (ALT), aspartate aminotransferase (AST), total protein (TP), albumin (Alb), globulin (Glo), γ-glutamyl transpeptidase (GGT), total bilirubin (TBIL), indirect bilirubin (IBIL), direct bilirubin (DBIL), total cholesterol (TC), high-density lipoprotein cholesterol (HDL), low-density lipoprotein cholesterol (LDL), triglycerides (TG), serum creatinine (SCr), and UA, were tested from blood specimens collected from anterior elbow veins. Urine pH (UpH) was acquired from urinalysis, which can indicate the crystal type of KSs (14). Estimated glomerular filtration rate (eGFR) was calculated using the Modification of Diet in Renal Disease (MDRD) Study China equation as follows: *eGFR* = 175 × *SCr*^−1.234^ × *age*^−0.179^ × 0.79(*if women*) (15). The SCr is in mg/dl unit.

### Statistical Analyses

Logistic regression models were used to assess the association of UA level with KS. The odds ratio (*OR*) and 95% *CI* were calculated taking per 50 μmol/L UA as a unit. Models were sequentially adjusted by age and sex (model 1), plus obesity (classified based on BMI according to the recommendation defined by the Working Group on Obesity in China: underweight, BMI ≤ 18.5 kg/m^2^; normal weight, BMI of 18.5 to ≤ 24 kg/m^2^; overweight, BMI of 24 to ≤ 28 kg/m^2^; and obese, BMI > 28 kg/m^2^) (16), diabetes (present/absent), CHD (present/absent), systolic blood pressure (SBP), diastolic blood pressure (DBP) (model 2), plus ALT, AST, Alb, Glo, GGT, eGFR, IBIL, DBIL, Glu, UpH, HDL, LDL, and TG (model 3). Model 3 was the primary model.

Restricted cubic splines with 5 knots (5th, 27.5th, 50th, 72.5th, and 95th percentiles of UA distribution) were used to determine the dose-response relationship between UA level and KS presence. The relationship was evaluated in different sex and age (<30, 30~50, ≥50) groups. The incidence rates of KS in the participants with stratified UA levels (<150, 150~249, 250~349, 350~449, 450~549, 550~649, 650~749, and ≥750 μmol/L for men and <150, 150~249, 250~349, 350~449, 450~549, 550~649, and ≥650 μmol/L for women) were calculated. The *OR* for KS in each stratified UA level was quantified by setting the group with the lowest risk as the reference. The trend in characteristics of participants with increasing UA levels was assessed through the logistic regression. The median values for continuous variables or proportions for categorical variables replace the original values for the participants in each category of UA level to compute the *p* for trend.

Subgroup analyses were conducted for men and women, respectively, by the following variables: obesity (underweight, normal weight, or overweight), hypertension (present, absent), diabetes (present, absent), CHD (present, absent), Alb (<35, 35~50, ≥50 g/L), TP (<60,60~80, ≥80 g/L), TC (<3, 3~5.2, ≥5.2 mmol/L), HDL (<1.0, ≥1.0 mmol/L), LDL (<3.4, ≥3.4 mmol/L), TG (<1.7, ≥1.7 mmol/L), eGFR (<90, 90~119, ≥120 ml/min/1.73 m^2^), Glu (<6.1, ≥6.1 mmol/L), and UpH (<5.5, ≥5.5). Mean differences (MDs) were calculated for each subgroup applying the bootstrap method to derive 95% *CI*s based on 100 bootstrap samples. In addition, the tests for interaction across subgroups were performed using the Wald test. A sensitivity analysis was conducted when the participants with missing data were included by comparing the effect sizes. R software (version 4.0.3) was used for the statistical analyses. Three R packages were used: dplyr, rms, and scales. All the values of *p* were two-tailed and *p* <0.05 was considered statistically significant.

## Results

### Baseline Characteristics of Participants With or Without KS

Among 82,017 participants (aged 18~99 years) included in the final analysis, 9,435 participants (11.5%) are diagnosed as KS based on the ultrasound examination. The mean age is 41.93 ± 12.88 years. A proportion of 56.3% individuals is male. The mean UA level for overall participants is 341.77 ± 95.51 μmol/L. The participants with KS have a much higher UA level than the participants without KS [mean UA level, 369.91 vs. 338.11 μmol/L; MD 31.96 [95% *CI*, 29.61~34.28] μmol/L) (Table 1). The basic characteristics for participants with or without KS are shown in (Table 1). The *OR* of per 50 μmol/L UA for KS is 1.179 (95% *CI*, 1.166~1.192) (Supplementary Table 1). The *OR*s for KS are 1.094, 1.079, and 1.072 after adjusting for model 1, model 2, and model 3, showing a stable relationship between UA and KS.

**Table 1:** Basic characteristics of included participants with or without kidney stone (KS).

**Variables**	**All Participants** **(***n*** = 82,017)**	**Participants with KS^a^** **(***n*** = 9,435)**	**Participants without KS^a^** **(***n*** = 72,582)**	***P-*** **value**
Age, y	41.93 ± 12.88	44.53 ± 12.41	41.59 ± 12.90	<0.001
Male (%)	46,201 (56.3)	6,831 (72.4)	39,370 (54.2)	<0.001
BMI, kg/m^2^^b^	23.62 ± 3.38	24.34 ± 3.31	23.52 ± 3.38	<0.001
Obesity (%)^c^				<0.001
Underweight (<18.5 kg/m^2^)	4,023 (4.9)	292 (3.1)	3,731 (5.1)	
Normal weight (18.5–23.9 kg/m^2^)	41,456 (50.5)	4,042 (42.8)	37,414 (51.5)	
Overweight (24–27.9 kg/m^2^)	28,022 (34.2)	3,750 (39.7)	24,272 (33.4)	
Obese (≥28 kg/m^2^)	8,516 (10.4)	1,351 (14.3)	7,165 (9.9)	
Hypertension present (%)	7,363 (9.0)	1,295 (13.7)	6,068 (8.4)	<0.001
Diabetes present (%)	2,103 (2.6)	357 (3.8)	1,746 (2.4)	<0.001
Coronary heart disease present (%)	472 (0.6)	80 (0.8)	392 (0.5)	<0.001
SBP, mmHg	123.98 ± 18.09	127.64 ± 18.82	123.51 ± 17.93	<0.001
DBP, mmHg	75.99 ± 12.09	78.74 ± 12.65	75.63 ± 11.97	<0.001
Glu, mmol/L	5.33 ± 1.11	5.48 ± 1.27	5.31 ± 1.09	<0.001
ALT, U/L	23.46 ± 22.63	26.08 ± 23.92	23.13 ± 22.44	<0.001
AST, U/L	22.01 ± 12.86	23.12 ± 13.72	21.87 ± 12.74	<0.001
TP, g/L	76.02 ± 3.91	75.90 ± 3.94	76.04 ± 3.91	0.001
Alb, g/L	46.13 ± 2.57	46.10 ± 2.58	46.13 ± 2.57	0.298
Glo, g/L	29.90 ± 3.54	29.80 ± 3.55	29.91 ± 3.54	0.004
GGT, U/L	31.22 ± 34.88	36.95 ± 38.28	30.48 ± 34.35	<0.001
TBIL, μmol/L	13.65 ± 5.45	13.94 ± 5.29	13.61 ± 5.47	<0.001
IBIL, μmol/L	9.97 ± 4.05	10.21 ± 3.95	9.94 ± 4.06	<0.001
DBIL, μmol/L	3.68 ± 1.72	3.73 ± 1.53	3.67 ± 1.75	0.004
TC, mmol/L	4.54 ± 0.87	4.63 ± 0.89	4.53 ± 0.86	<0.001
HDL, mmol/L	1.29 ± 0.31	1.23 ± 0.29	1.29 ± 0.31	<0.001
LDL, mmol/L	2.73 ± 0.75	2.80 ± 0.76	2.72 ± 0.74	<0.001
TG, mmol/L	1.47 ± 1.26	1.69 ± 1.44	1.44 ± 1.23	<0.001
SCr, μmol/L	73.83 ± 18.72	78.92 ± 19.92	73.17 ± 18.45	<0.001
eGFR, mL/min/1.73m^2^^d^	107.43 ± 22.72	101.99 ± 22.63	108.14 ± 22.63	<0.001
UA, μmol/L^e^	341.77 ± 95.51	369.91 ± 102.87	338.11 ± 93.90	<0.001
UpH	6.12 ± 0.65	6.09 ± 0.64	6.12 ± 0.65	<0.001

*KS, kidney stone; BMI, body mass index; SBP, systolic blood pressure; DBP, diastolic blood pressure; Glu, fasting glucose; ALT, alanine aminotransferase; AST, aspartate aminotransferase; TP, total protein; Alb, albumin; Glo, globulin; GGT, γ-glutamyl transpeptidase; TBIL, total bilirubin; IBIL, indirect bilirubin; DBIL, direct bilirubin; TC, total cholesterol; HDL, high-density lipoprotein cholesterol; LDL, low-density lipoprotein cholesterol; TG, triglycerides; SCr, serum creatinine; eGFR, estimated glomerular filtration rate; UA, uric acid; UpH, Urine pH*.

*No. of participants have missing data for the variables: sex, n = 1; BMI, n = 4,839; blood pressure, n = 3,615; Glu, n = 3,261; ALT, n = 1,498; AST, n = 2,646; TP, Alb, n = 9,944; Glo, n = 9,969; GGT, n = 8,338; SCr, n = 1,478; TBIL, n = 7,053; IBIL, n = 7,998; DBIL, 7,899; TC, TG, n = 3,344; HDL, LDL n = 3,901; UA, n = 3,349; UpH, n = 3,683*.

a *Structures reported by ultrasonography examination to be strong echoes in renal sinus with acoustic shadows in posterior or with comet tail signs were regarded as KS regardless of the size*.

b *Calculated as weight in kilograms divided by height in meters squared*.

c *Classified according to the recommendation defined by Working Group on Obesity in China*.

d *Calculated using the CKD-EPI equation (Details can be found in Methods section)*.

e *Mean difference (95% CI) was 31.96 (29.61~34.28) μmol/L (details can be found in Methods section)*.

### Correlation Between OR for KS and UA Level

**Figure 1:**
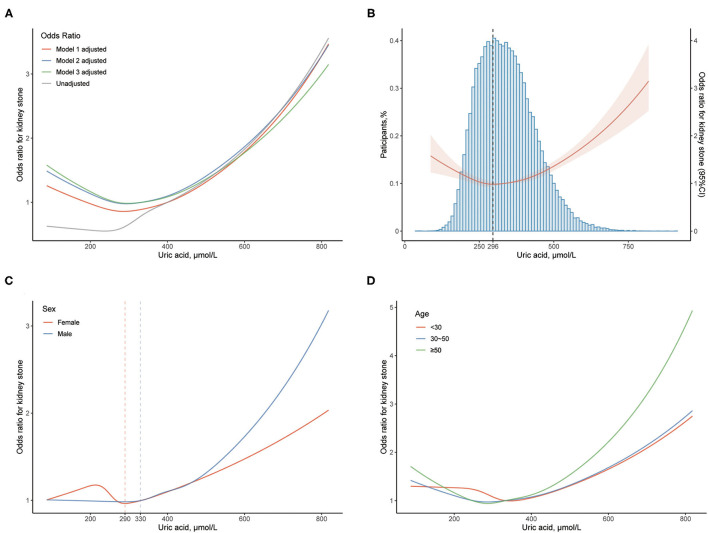
Association between uric acid (UA) level and kidney stone (KS). **(A)** The odds ratio (OR) is indicated by the curves with multivariable adjustment. **(B)** The *OR* with 95% *CI* is indicated by the red line and shade; blue histogram implies the proportion of the participants. The dashed line reveals the UA level (296 μmol/L) where the odds of KS start to increase. **(C)** The OR is stratified by sex. The dashed lines indicate the UA levels (330 and 290 μmol/L) where the odds of KS start to increase in male and female, respectively. **(D)** The *OR* is stratified by age. The *OR* with 95% *CI* of KS is calculated by taking UA level of 400 μmol/L as reference. Models: see Methods-Statistical analyses Section for descriptions of models 1, 2, and 3. Model 3 is applied unless demonstrated.

We further explore the correlative pattern between *OR* for KS and UA levels. The restricted cubic splines illustrate that the correlative curves for UA level and *OR* of KS are similar after the adjustment of different models (Figure 1A). When the UA level is >296 μmol/L, the *OR* of KS starts to increase with UA level (Figure 1B). However, the *OR* increases more quickly in men (from UA 330 μmol/L) than in women (from UA 290 μmol/L, Figure 1C). Each 50 μmol/L elevation of UA level increases the risk of KS formation by 10.7% above the UA level of 330 μmol/L in men. The participants aged over 50 years have higher odds of KS than those aged under 50 years (Figure 1D).

### Stratified Analyses by UA Level and Sex

Based on the difference between sexes, further analyses focus on sex disparities. Taking the UA level 250~349 μmol/L as the reference in the male population, we found that the *OR*s are 1.157, 1.478, 2.025, 2.525, and 3.644 for UA levels 350~449, 450~549, 550~649, 650~749, and ≥750 μmol/L after adjusted by age, which show a significantly growing trend (Table 2). In female individuals, an increasing trend of *OR* of KS 1.029, 1.276, 1.560, and 2.002 after adjusted by age is also observed by taking the UA level 250~349 μmol/L as reference. However, the tendency is not of statistical significance (Table 2). The ORs adjusted by age, model 2, or model 3 indicate similar results. The characteristics of men with stratified UA levels demonstrate significant trends with *p* for trend <0.05 (Supplementary Table 2). However, the tendency is not observed in women (Supplementary Table 3). These results indicate that UA possesses the capability to stratify participants with different characteristics in men but not in women.

**Table 2:** Odds ratio (*OR*) for KS stratified by UA level and sex.

**Stratified Uric Acid Level (μmol/L)**	**Participants with KS/Total Participants**	**Incidence Rate (%)**	**Adjusted Odds Ratio (95%CI)**					
			**Age-Adjusted**	***P*** **-value**	**Model 2^a^**	***P-*** **value**	**Model 3^a^**	***P*** **-value**
**Male**
<150	1/22	4.55	0.318 (0.018~1.524)	0.263	0.315 (0.018~1.512)	0.260	0.328 (0.018~1.574)	0.277
150~249	158/1,170	13.50	0.993 (0.830~1.180)	0.937	1.006 (0.841~1.196)	0.946	1.021 (0.853~1.215)	0.818
250~349	1,736/13,415	12.94	Ref.		Ref.		Ref.	
350~449	3,017/21,177	14.25	1.157 (1.086~1.233)	<0.001	1.118 (1.048~1.193)	<0.001	1.092 (1.023~1.166)	0.001
450~549	1,449/8,358	17.34	1.478 (1.369~1.595)	<0.001	1.379 (1.275~1.492)	<0.001	1.313 (1.209~1.425)	<0.001
550~649	385/1,740	22.13	2.025 (1.786~2.291)	<0.001	1.839 (1.618~2.086)	<0.001	1.709 (1.496~1.949)	<0.001
650~749	71/277	25.63	2.525 (1.905~3.308)	<0.001	2.269 (1.710~2.978)	<0.001	2.029 (1.521~2.677)	<0.001
≥750	14/42	33.33	3.644 (1.855~6.840)	<0.001	3.195 (1.624~6.007)	<0.001	2.820 (1.429~5.322)	0.002
**Female**
<150	17/254	6.69	0.996 (0.584~1.586)	0.989	0.998 (0.585~1.589)	0.993	0.960 (0.562~1.533)	0.874
150~249	993/1,2894	7.70	1.161 (1.065~1.266)	0.001	1.167 (1.069~1.274)	0.001	1.154 (1.054~1.264)	0.002
250~349	1,267/18,482	6.86	Ref.		Ref.		Ref.	
350~449	278/3,700	7.51	1.029 (0.897~1.177)	0.681	1.018 (0.886~1.166)	0.797	1.021 (0.887~1.172)	0.770
450~549	42/430	9.77	1.276 (0.909~1.746)	0.143	1.242 (0.882~1.703)	0.196	1.249 (0.885~1.719)	0.188
550~649	6/49	12.24	1.560 (0.592~3.419)	0.312	1.520 (0.576~3.338)	0.341	1.553 (0.587~3.428)	0.319
≥650	1/7	14.29	2.002 (0.105~11.936)	0.523	1.956 (0.103~11.606)	0.537	2.168 (0.114~12.915)	0.477

*UA, uric acid; KS, kidney stone; CI, confidence interval*.

a *Models: see Methods-Statistical analyses section for descriptions of models 2 and 3*.

### Subgroup Analyses on the Association Between UA Levels and KS

**Figure 2:**
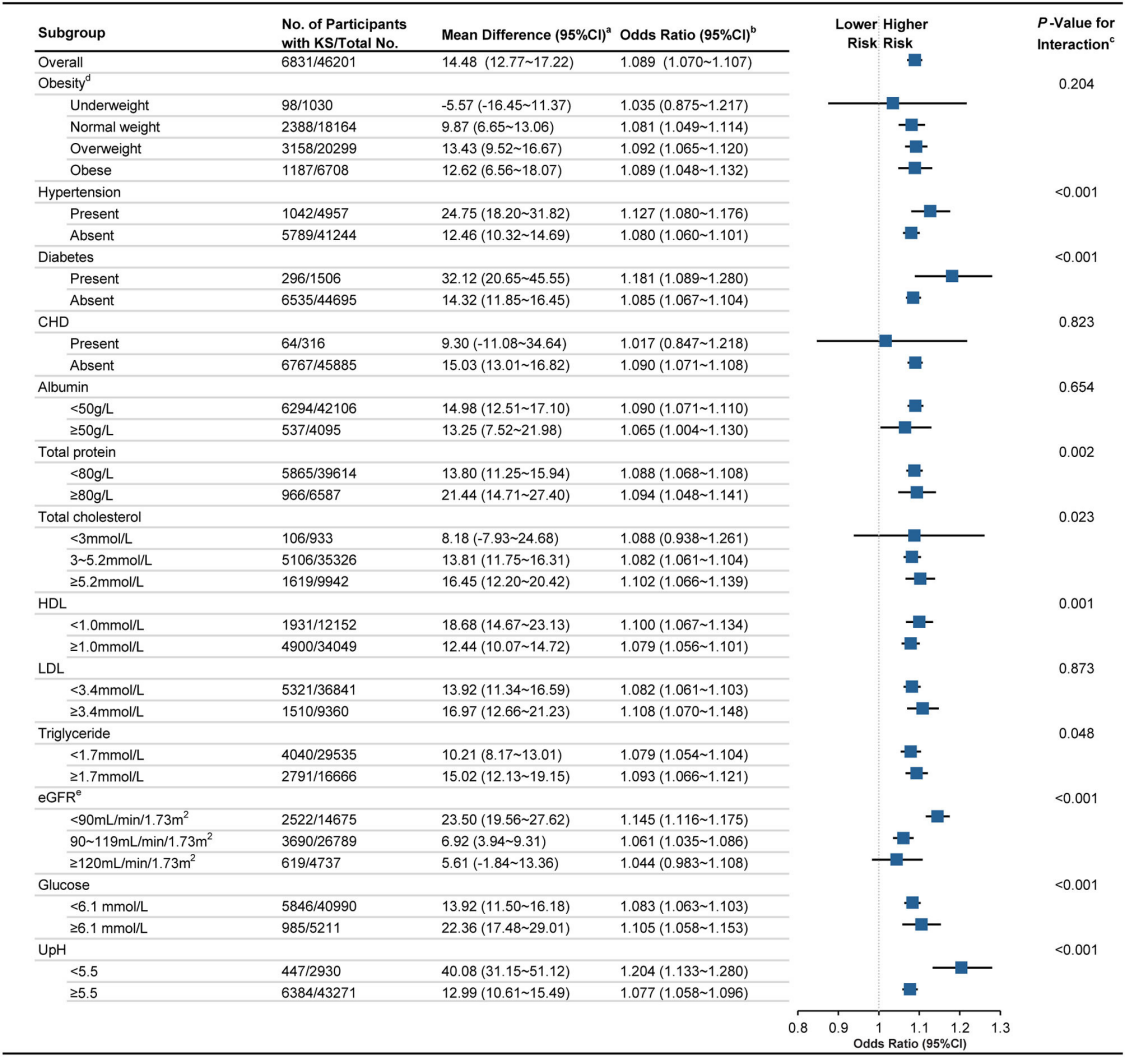
Association between UA level and KS in male population among different subgroups. KS, kidney stone; CI, confidence interval; CHD coronary heart disease; HDL, high-density lipoprotein cholesterol; LDL, low-density lipoprotein cholesterol; eGFR, estimated glomerular filtration rate; UpH, Urine pH. ^a^ Calculated applying bootstrap method. Details can be found in Methods-Statistical analyses Section. ^b^ Calculated applying model 3 (as shown in Methods-Statistical analyses Section for descriptions of model 3) and taking per 50 μmol/L UA as a unit. ^c^ Calculated by applying Wald test. ^d^ Classified according to the recommendation defined by Working Group on Obesity in China. ^e^ Calculated using the CKD–EPI equation. Details can be found in Methods Section.

**Figure 3:**
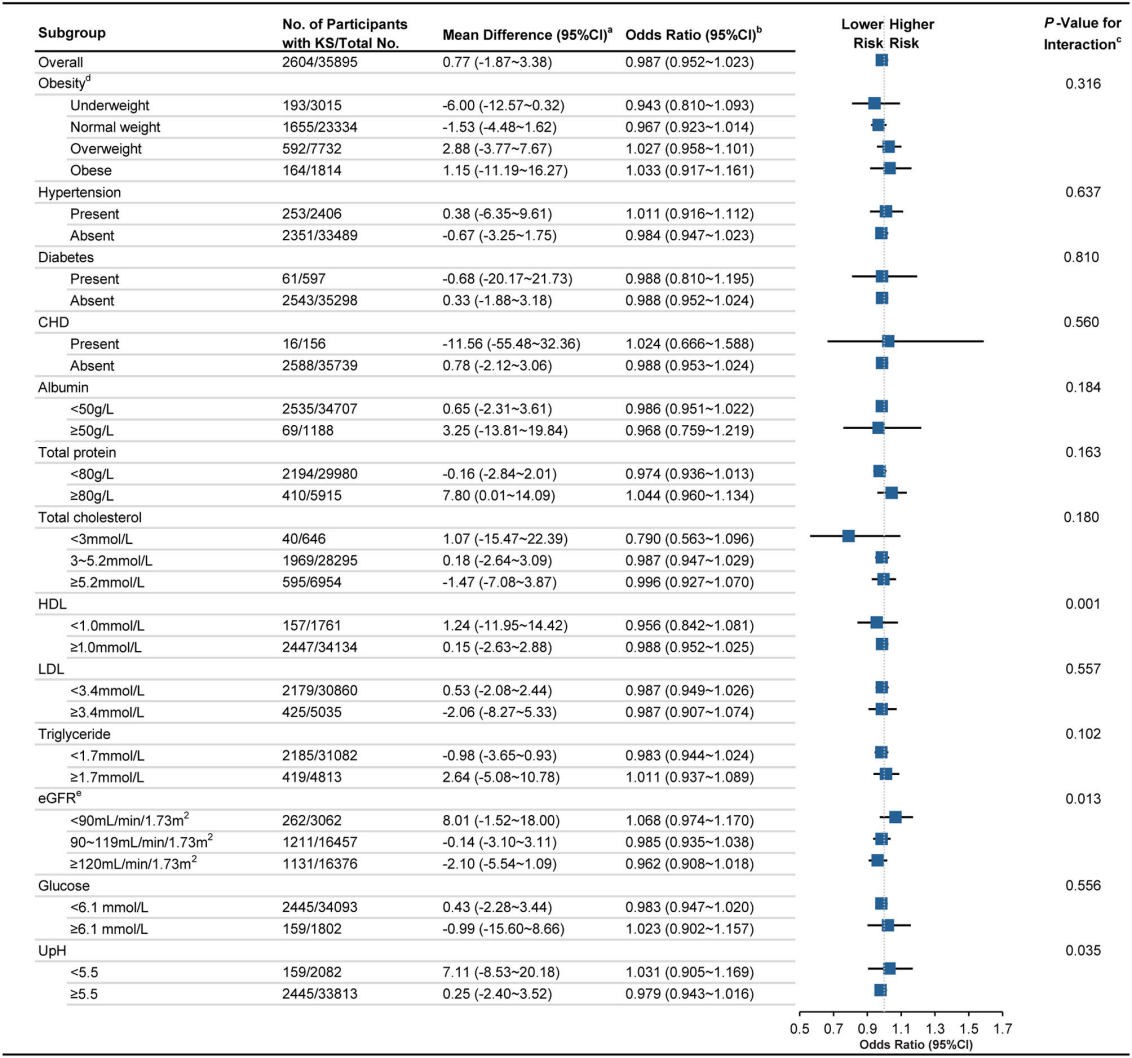
Association between UA level and KS in female population among different subgroups. KS, kidney stone; *CI*, confidence interval; CHD coronary heart disease; HDL, high-density lipoprotein cholesterol; LDL, low-density lipoprotein cholesterol; eGFR, estimated glomerular filtration rate; UpH, Urine pH. ^a^ Calculated applying bootstrap method. Details can be found in Methods-Statistical analyses Section. ^b^ Calculated applying model 3 (as shown in Methods-Statistical Analyses Section for descriptions of model 3) and taking per 50 μmol/L UA as a unit. ^c^ Calculated by applying Wald test. ^d^ Classified according to the recommendation defined by Working Group on Obesity in China. ^e^ Calculated using the CKD–EPI equation. Details can be found in Methods Section.

In subgroup analysis for men, it was implied that the association between UA levels and KS is significant for participants presenting hypertension [adjusted *OR*, 1.127 (95% *CI*, 1.080~1.176); MD, 24.75 (95% *CI*, 18.20~31.82); *p* for interaction, <0.001], diabetes [adjusted *OR*, 1.181 (1.089~1.128); MD, 32.12 (20.65~45.55); *p* <0.001], high TP [adjusted *OR*, 1.094 (1.048~1.132); MD, 21.44 (14.71~27.40); *p* 0.002], high TC [adjusted *OR*, 1.102 (1.066~1.139); MD, 16.45 (12.20~20.42); *p* <0.023], low HDL [adjusted *OR*, 1.100 (1.067~1.134); MD, 12.44 (10.07~14.72); *p* <0.001], high TG [adjusted *OR*, 1.093 (1.066~1.121); MD, 15.02 (12.13~19.15); *p* <0.048], low eGFR [adjusted *OR*, 1.145 (1.116~1.175); MD, 23.50 (19.56~27.62); *p* <0.001], high Glu [adjusted *OR*, 1.105 (1.058~1.153); MD, 22.36 (17.48~29.01); *p* <0.001], and low UpH [adjusted *OR*, 1.204 (1.133~1.280); MD, 40.08 (31.15~51.12); *p* <0.001]. However, the association between UA level and KS is not significant for the men presenting underweight [adjusted *OR*, 1.035 (0.875~1.217); MD, −5.57 (−16.45~11.37)], CHD [adjusted *OR*, 1.017 (0.847~1.218); MD, 9.30 (−11.08~34.64)], low TC [adjusted *OR*, 1.088 (0.938~1.261); MD, 8.18 (−7.93~24.68)], and high eGFR [adjusted *OR*, 1.044 (0.983~1.108); MD, 5.61 (−1.84~13.36), Figure 2]. However, no significant association between UA and KS is observed in female in all subgroups (Figure 3).

### Sensitivity Analysis

The sensitivity analysis is performed between the population with and without missing data (Supplementary Table 4). In the population without missing data, the *OR*s of KS are 1.179, 1.094, 1.079, and 1.073, respectively, after unadjusted, model 1 adjusted, model 2 adjusted, and model 3 adjusted. In the population with missing data, the *OR*s of KS are 1.184, 1.098, 1.084, and 1.073, respectively. The mean difference of UA is 31.96 (95% *CI*, 29.61~34.28) μmol/L and 32.71 (31.00~34.16) μmol/L for participants without and with missing data. Both populations indicate non-linear trends for the association between UA level and OR of KS. These results demonstrate the statistical stability after excluding participants with missing data.

## DISCUSSION

Among 82,017 Chinese participants from the chronic diseases cohort who had undergone physical examination in 2017, it was discovered that increased UA levels were associated with a higher risk of KS in a dose-response manner in men above the UA level of 330 μmol/L. However, no significant increased risk of KS parallel to UA level is observed in women. These results indicate that the risk of KS starts at a lower UA level than the normal upper limit. Besides, for the men presenting underweight, low level of TC, or high level of eGFR, the association between UA level and KS development is no longer significant.

To the best of our knowledge, this was the first study to investigate the association between serum UA levels and KS development in the Chinese population. The prevalence of urolithiasis presented great variations in different geographic locations in Asia. In West Asia, Southeast Asia, and South Asia, the prevalence was reported to be 5–19.1%, however, it was only 1–8% in East Asia and North Asia (17). The prevalence of KS showed similar heterogeneity in different regions in China with a prevalence of 11.6% in the South (18). Moreover, the highest prevalence in China was 13.69% in Sichuan Province, which was geographically close to Hubei Province (19). Hence, it was of importance to obtain a further understanding of the KS development in Chinese populations.

Previous studies have demonstrated the association between hyperuricemia and urolithiasis using the data from the National Health and Nutrition Examination Survey 2007–2008 and the data from Kangbuk Samsung Health Study (9, 20). Convincing evidence includes that allopurinol and other urate-lowering therapies can reduce the recurrence rate of KS (21). Moreover, clinical and laboratory experiments demonstrate that oxygen species and oxidative stress followed by an inflammatory immune response are widely acknowledged to promote the KS initiation (22, 23). UA and its monoanionic urate increase oxidative damage and inactivate enzymes that are sensitive to the oxidative stress inducing kidney injury, despite that serum UA plays a role of a potent antioxidant under physiological circumstances (24). Recent research described the response of renal tubular cells to high doses of UA, which partially explained that hyperuricemia can induce mixed (UA and calcium oxide) KS (25). Since UA is reported to be responsible for KS of various components, controlling the level of UA may provide the key point to prevent KS from formation. Further research on the mechanism underlying the relation between serum UA levels and urolithiasis is required.

We found that UA differentially promoted the KS formation in male and female. One possible explanation for the sex disparities in the association between serum UA levels and KS is sex steroid differences that result in distinct states of oxidative stress, inflammation, and immune response between men and women (26). Testosterone was stated to be a predisposing factor for kidney disease, while E2 has been proved to harbor protective effects in women (27). Mechanically, testosterone increases an alpha-enolase expression on the surface of renal tubular cells, which contributes to the crystal-cell adhesion (28). Exogenous testosterone increases the risk of stone events, while androgen deprivation therapy and finasteride reduce the risk of renal calculi (29, 30). Estrogen may lower the urinary calcium and calcium oxalate saturation, decrease surface expression of calcium oxide crystal receptors, alleviate intracellular metabolism, and promote cell proliferation and tissue healing to prevent the KS formation (31, 32). A recent comparative study suggested that postmenopausal status was associated with a higher risk of KS development (33). Moreover, menopause was independently associated with higher UA levels that can be reduced by estrogen use (34). More studies are needed to unfold the mechanism attenuating the association between UA levels and urolithiasis in women. Sex disparities should be taken into consideration when preventing KS from formation according to UA levels.

In the men, who present underweight, low TC level, or high eGFR level, the KS development is not significantly associated with UA. The high eGFR level calculated from creatinine may result from muscle loss, suggesting a malnutrition status (35). We speculate that malnutrition status attenuates the association between UA and KS, namely, adequate nutrition status may be necessary for the KS development. Due to the small population of those presenting low Alb level (<35 g/L, *n* = 50), and low TP level (<60 g/L, *n* = 17) which also reflect a malnutrition status, subgroup analyses are not conducted for these two factors. Dietary factors have the potential of influencing the microbiota composition associated with KS formation, nevertheless, the correlation between malnutrition and KS remains to be elaborated (36).

We adjust UpH in the model since different stone types can be formed under different UpH levels, which previous studies have not contained. Alkaline UpH is favorable for the crystallization of calcium and phosphate containing stone, while acidic urine UpH promotes the formation of calcium oxalate, UA, and cystine stone (37). In this study, the UpH is applied to reflect the stone types and urine chemistry. After adjusting urine pH, UA level is still significantly associated with KS development in men.

Besides KS, UA causes many other diseases with thresholds under the normal upper limit (38). Maintaining the serum UA levels below the saturation point for monosodium urate (≤360 μmol/L) is recommended from the perspective of gout prevention (39). Hyperuricemia proves to be an independent risk factor for the development of type 2 diabetes and predicts hypertension (40). Increased UA levels are associated with the risk of congestive heart failure, stroke, and atrial fibrillation (41). However, from the perspective of preventing hypertension, the cutoff value for UA levels is 345 μmol/L for men and 274 μmol/L for women (42). In this study, concerned with urolithiasis development, the cutoff value for UA levels is suggested to be 330 μmol/L for men. We recommend that the disease- and gender-specific cutoff values for UA levels should be raised for precise prevention and treatment.

## Limitations

This study still has several limitations. First, the study findings are observational without establishing causality. Instead, we construct a dose-response relationship between KS and UA using the restricted cubic splines method. Second, urolithiasis is diagnosed by US rather than non-contrast CT. As a radiation-free and low-cost imaging modality, US is strongly recommended for screening in a large population (43). Third, missing data were handled by deleting instead of applying statistic methods. It is based on the consideration that the data are not followed a “missing at random” pattern because a participant is usually absent from a full set of tests. In the sensitivity analysis, no significant difference was observed after excluding the participants with missing data. Fourth, no dietary information was obtained to adjust the effect size. Nevertheless, compared with the gene effect, dietary factor explains very little variation in UA levels (44). Fifth, we lacked the history of gout and menopause, although gout and menopause were reported to be involved in KS formation. Further studies concerning the gout and menopause were warranted.

## CONCLUSION

Among Chinese adults, increased UA level is associated with a higher risk of KS in a dose-response manner in men above the UA level of 330 μmol/L. However, no increased risk of urolithiasis parallel to UA level is observed in women. We suggest a cutoff UA level lower than the upper normal limit to prevent KS formation. These findings will contribute to unraveling the pathophysiology of urolithiasis and further cast light on the prevention of KS development and recurrence.

## Data Availability Statement

The original contributions presented in the study are included in the article/Supplementary Materials, further inquiries can be directed to the corresponding authors.

## Ethics Statement

Written informed consent was obtained from the individual(s) for the publication of any potentially identifiable images or data included in this article.

## Author Contributions

X-ML, WG, and CL had full access to all the data in the study, take responsibility for the integrity of the data, and the accuracy of the data analysis. J-ZX, J-LL, LH, X-ML, WG, and CL contributed to conceptualization. J-ZX, J-LL, and LH contributed to formal analysis, methodology, software, statistical analysis, and writing—original draft. YX, Z-CW, Q-DX, X-YQ, Y-YY, S-YH, S-GW, X-ML, WG, and CL contributed to validation and visualization. S-GW, X-ML, WG, and CL contributed to resources, supervision, and writing—review and editing. All authors contributed to data curation and project administration. All authors contributed to the article and approved the submitted version.

## Conflict of Interest

The authors declare that the research was conducted in the absence of any commercial or financial relationships that could be construed as a potential conflict of interest.

## Publisher's Note

All claims expressed in this article are solely those of the authors and do not necessarily represent those of their affiliated organizations, or those of the publisher, the editors and the reviewers. Any product that may be evaluated in this article, or claim that may be made by its manufacturer, is not guaranteed or endorsed by the publisher.

## Abbreviations

KS: kidney stone
UA: uric acid
BMI: body mass index
SBP: systolic blood pressure
DBP: diastolic blood pressure
Glu: fasting glucose
ALT: alanine aminotransferase
AST: aspartate aminotransferase
TP: total protein
Alb: albumin
Glo: globulin
GGT: γ-glutamyl transpeptidase
TBIL: total bilirubin
IBIL: indirect bilirubin
DBIL: direct bilirubin
TC: total cholesterol
HDL: high-density lipoprotein cholesterol
LDL: low-density lipoprotein cholesterol
TG: triglycerides
SCr: serum creatinine
eGFR: estimated glomerular filtration rate
UpH: Urine pH
*OR*: odds ratio
MD: mean difference
*CI*: confidence interval.
