# Supplementary material for: Sex Disparities in the Association of Serum Uric Acid With Kidney Stone: A Cross-Sectional Study in China
Source: Front Med (Lausanne). 2022 Feb 9;9:774351. doi: 10.3389/fmed.2022.774351 (PMC8864179; doi:10.3389/fmed.2022.774351)
Supplement: Supplementary file 1 [file Data_Sheet_1.docx]

**eTable 1. Association between UA level and KS using an extended model approach**

|  | Odds ratio (95%CI) of UA (per 50μmol/L) ^b^ | *P*-value |
| --- | --- | --- |
|  |  |  |
| Unadjusted | 1.179(1.166~1.192) | < 0.001 |
| Model 1 ^a^ | 1.094(1.079~1.108) | < 0.001 |
| Model 2 ^a^ | 1.079(1.064~1.094) | < 0.001 |
| Model 3 ^a^ | 1.072(1.055~1.088) | < 0.001 |

See the Table 1 footnote for the criteria of diagnosis for urolithiasis.

^a^ Models: Model 1: adjusted for age and sex.

Model 2: Model 1 plus obesity (classified based on BMI according to the recommendation defined by Working Group on Obesity in China), diabetes (present/absent), coronary heart disease (present/absent), SBP, DBP.

Model 3: Model 2 plus ALT, AST, Alb, Glo, GGT, eGFR, IBIL, DBIL, Glu, UpH, HDL, LDL, and TG.

^b^ Odds ratio (OR) and 95% confidence interval (CI) of UA were calculated taking per 50 μmol/L UA as a unit.

Abbreviations: UA, serum uric acid; KS, kidney stone; BMI, body mass index; SBP, systolic blood pressure; DBP, diastolic blood pressure; ALT, alanine aminotransferase; AST, aspartate aminotransferase; Alb, albumin; Glo, globulin; GGT, γ-glutamyl transpeptidase; eGFR, estimated glomerular filtration rate; IBIL, indirect bilirubin; DBIL, direct bilirubin; Glu, fasting glucose; UpH, Urine pH; HDL, high-density lipoprotein cholesterol; LDL, low-density lipoprotein cholesterol; TG, triglyceride.

**eTable 2. Characteristics of male participants with stratified UA level**

| Variables | Overall | Stratified UA group | | | | | | | | *P* for trend |
| --- | --- | --- | --- | --- | --- | --- | --- | --- | --- | --- |
|  |  | <150 | 150~249 | 250~349 | 350~449 | 450~549 | 550~649 | 650~749 | ≥750 |  |
| Age, y | 42.63±12.74 | 44.23±14.09 | 47.60±13.55 | 44.12±13.09 | 42.06±12.57 | 41.47±12.17 | 40.88±12.26 | 39.27±11.92 | 39.69±13.45 | <0.001 |
| BMI, kg/m^2 a^ | 24.65±3.23 | 24.23±3.63 | 23.09±3.07 | 23.60±3.02 | 24.73±3.08 | 25.85±3.20 | 26.61±3.30 | 26.83±3.72 | 27.27±3.73 | <0.001 |
| Obesity (%) ^b^ |  |  |  |  |  |  |  |  |  |  |
| Underweight | 1030(2.2) | 0(0.0) | 72(6.2) | 515(3.8) | 374(1.8) | 62(0.7) | 6(0.3) | 1(0.4) | 0(0.0) | 0.548 |
| Normal weight | 18164(39.3) | 13(59.1) | 654(55.9) | 6876(51.3) | 8048(38.0) | 2175(26.0) | 331(19.0) | 59(21.3) | 8(19.0) | <0.001 |
| Overweight | 20299(43.9) | 5(22.7) | 371(31.7) | 4981(37.1) | 9774(46.2) | 4178(50.0) | 848(48.7) | 123(44.4) | 19(45.2) | <0.001 |
| Obese | 6708(14.5) | 4(18.2) | 73(6.2) | 1043(7.8) | 2981(14.1) | 1943(23.2) | 555(31.9) | 94(33.9) | 15(35.7) | <0.001 |
| Hypertension present (%) | 4957(10.7) | 1(4.5) | 124(10.6) | 1242(9.3) | 2205(10.4) | 1054(12.6) | 277(15.9) | 47(17.0) | 7(16.7) | <0.001 |
| Diabetes (%) | 1506(3.3) | 2(9.1) | 91(7.8) | 582(4.3) | 587(2.8) | 198(2.4) | 35(2.0) | 9(3.2) | 2(4.8) | <0.001 |
| CHD (%) | 316(0.7) | 0(0.0) | 11(0.9) | 98(0.7) | 132(0.6) | 60(0.7) | 14(0.8) | 1(0.4) | 0(0.0) | 0.905 |
| SBP, mmHg | 127.68±17.41 | 131.32±15.94 | 126.64±17.94 | 125.59±17.25 | 127.47±17.15 | 130.22±17.38 | 133.39±18.00 | 135.25±19.82 | 135.43±20.25 | <0.001 |
| DBP, mmHg | 79.06±11.91 | 78.41±11.28 | 77.58±11.56 | 77.34±11.50 | 78.98±11.79 | 81.12±12.07 | 83.32±12.67 | 84.17±13.22 | 86.10±16.25 | <0.001 |
| ALT, U/L | 28.63±25.74 | 22.73±14.53 | 22.70±16.53 | 24.27±20.90 | 28.65±27.59 | 33.57±25.77 | 39.59±29.62 | 42.15±30.98 | 52.26±65.65 | <0.001 |
| AST, U/L | 23.69±14.49 | 20.82±6.43 | 22.15±12.08 | 22.14±12.22 | 23.58±15.23 | 25.47±15.39 | 28.27±15.07 | 29.05±18.02 | 36.14±31.45 | <0.001 |
| TP, g/L | 75.88±3.89 | 75.81±4.12 | 74.87±3.88 | 75.31±3.90 | 75.91±3.81 | 76.49±3.83 | 77.12±4.02 | 78.17±4.51 | 78.49±4.25 | <0.001 |
| Alb, g/L | 46.60±2.57 | 46.40±3.25 | 45.86±2.87 | 46.34±2.60 | 46.67±2.52 | 46.82±2.50 | 47.01±2.65 | 47.15±2.84 | 47.44±2.83 | <0.001 |
| Glo, g/L | 29.28±3.49 | 29.42±4.08 | 29.01±3.64 | 28.97±3.55 | 29.24±3.42 | 29.67±3.45 | 30.11±3.57 | 31.02±3.98 | 31.05±3.35 | <0.001 |
| GGT, U/L | 39.95±41.90 | 28.64±20.78 | 34.76±85.19 | 32.42±30.22 | 39.39±38.04 | 49.31±45.76 | 58.44±68.56 | 64.14±50.08 | 92.90±149.62 | <0.001 |
| TBIL, μmol/L | 14.71±5.81 | 14.34±4.42 | 14.78±5.46 | 14.77±5.91 | 14.78±5.86 | 14.56±5.57 | 14.42±5.93 | 13.62±4.71 | 13.18±4.89 | <0.001 |
| IBIL, μmol/L | 10.70±4.32 | 10.48±3.26 | 10.67±3.98 | 10.70±4.59 | 10.74±4.21 | 10.65±4.24 | 10.56±4.17 | 10.03±3.49 | 9.58±3.67 | 0.046 |
| DBIL, μmol/L | 4.02±1.90 | 3.86±1.43 | 4.12±1.85 | 4.07±1.63 | 4.04±2.12 | 3.91±1.54 | 3.86±2.51 | 3.59±1.43 | 3.60±1.57 | <0.001 |

eTable 2. Characteristics of male participants with stratified UA level (continued)

| Variables | Overall | Stratified UA group | | | | | | | | *P* for trend |
| --- | --- | --- | --- | --- | --- | --- | --- | --- | --- | --- |
|  |  | <150 | 150~249 | 250~349 | 350~449 | 450~549 | 550~649 | 650~749 | ≥750 |  |
| TC, mmol/L | 4.57±0.87 | 4.48±0.83 | 4.46±0.87 | 4.45±0.83 | 4.57±0.86 | 4.73±0.90 | 4.81±0.92 | 4.96±1.03 | 5.11±1.04 | <0.001 |
| HDL, mmol/L | 1.16±0.25 | 1.30±0.30 | 1.26±0.27 | 1.23±0.27 | 1.15±0.24 | 1.10±0.23 | 1.05±0.20 | 1.04±0.21 | 1.05±0.29 | <0.001 |
| LDL, mmol/L | 2.81±0.75 | 2.73±0.68 | 2.73±0.73 | 2.74±0.72 | 2.82±0.74 | 2.88±0.77 | 2.88±0.81 | 2.91±0.89 | 3.06±0.86 | <0.001 |
| TG, mmol/L | 1.74±1.46 | 1.34±0.65 | 1.30±1.01 | 1.39±1.09 | 1.73±1.41 | 2.17±1.75 | 2.55±1.94 | 2.93±2.75 | 2.80±2.00 | <0.001 |
| SCr, μmol/L | 84.40±17.17 | 79.27±20.79 | 77.08±11.61 | 81.14±13.52 | 84.39±12.43 | 88.19±14.72 | 92.34±19.15 | 107.70±129.53 | 103.05±34.07 | <0.001 |
| eGFR, mL/min/1.73m2 ^c^ | 98.36±17.56 | 109.64±27.96 | 107.86±19.96 | 102.18±17.20 | 98.13±16.59 | 93.74±17.41 | 89.90±18.70 | 84.72±21.65 | 82.66±22.04 | <0.001 |
| Glu, mmol/L | 5.45±1.25 | 5.93±1.86 | 6.10±2.44 | 5.52±1.46 | 5.41±1.09 | 5.38±0.96 | 5.44±1.05 | 5.61±1.95 | 5.55±1.46 | <0.001 |
| UpH | 6.07 ± 0.64 | 6.07 ± 0.60 | 6.16 ± 0.66 | 6.16 ± 0.66 | 6.08 ± 0.64 | 5.96 ± 0.61 | 5.86 ± 0.58 | 5.77 ± 0.51 | 5.65 ± 0.60 | <0.001 |

Abbreviations: UA, serum uric acid; BMI, body mass index; CHD, coronary heart disease; SBP, systolic blood pressure , DBP, diastolic blood pressure; ALT, alanine aminotransferase; AST, aspartate aminotransferase; TP, total protein; Alb, albumin; Glo, globulin; GGT, γ-glutamyl transpeptidase; TBIL, total bilirubin; IBIL, indirect bilirubin; DBIL, direct bilirubin; TC, total cholesterol; HDL, high-density lipoprotein cholesterol; LDL, low-density lipoprotein cholesterol; TG, triglycerides; SCr, serum creatinine; eGFR, estimated glomerular filtration rate; Glu, fasting glucose; UpH, Urine pH.

^a^ Calculated as weight in kilograms divided by height in meters squared.

^b^ Classified according to Asian-specific criteria.

^c^ Calculated using the CKD-EPI equation (Details can be found in Methods section).

**eTable 3. Characteristics of female participants with stratified UA level**

| Variables | Overall | Stratified UA group | | | | | | | *P* for trend |
| --- | --- | --- | --- | --- | --- | --- | --- | --- | --- |
|  |  | <150 | 150~249 | 250~349 | 350~449 | 450~549 | 550~649 | ≥650 |  |
| Age, y | 41.02±12.99 | 40.00±11.68 | 39.93±11.68 | 40.94±13.11 | 44.33±15.25 | 48.04±16.63 | 50.88±17.71 | 47.29±17.69 | 0.041 |
| BMI, kg/m^2 a^ | 22.29±3.10 | 20.94±2.58 | 21.46±2.65 | 22.45±3.05 | 24.04±3.58 | 25.65±3.95 | 25.32±3.77 | 23.89±3.96 | 0.862 |
| Obesity (%) ^b^ |  |  |  |  |  |  |  |  |  |
| Underweight | 2993(8.4) | 39(15.4) | 1470(11.4) | 1344(7.3) | 133(3.6) | 6(1.4) | 1(2.0) | 0(0.0) | 0.412 |
| Normal weight | 23292(65.0) | 182(71.7) | 9270(71.9) | 11877(64.3) | 1800(48.6) | 144(33.5) | 16(32.7) | 3(42.9) | 0.620 |
| Overweight | 7723(21.6) | 31(12.2) | 1904(14.8) | 4329(23.4) | 1264(34.2) | 172(40.0) | 20(40.8) | 3(42.9) | 0.953 |
| Obese | 1808(5.0) | 2(0.8) | 250(1.9) | 932(5.0) | 503(13.6) | 108(25.1) | 12(24.5) | 1(14.3) | 0.913 |
| Hypertension present (%) | 2406(6.7) | 8(3.1) | 486(3.8) | 1233(6.7) | 550(14.9) | 106(24.7) | 21(42.9) | 2(28.6) | 0.283 |
| Diabetes present (%) | 597(1.7) | 0(0.0) | 160(1.2) | 281(1.5) | 121(3.3) | 31(7.2) | 3(6.1) | 1(14.3) | 0.064 |
| CHD present (%) | 156(0.4) | 0(0.0) | 36(0.3) | 63(0.3) | 47(1.3) | 8(1.9) | 2(4.1) | 0(0.0) | 0.083 |
| SBP, mmHg | 119.22±17.83 | 116.33±17.36 | 116.47±16.12 | 119.59±17.84 | 125.33±20.20 | 132.72±21.94 | 135.20±23.02 | 138.57±16.84 | 0.317 |
| DBP, mmHg | 72.03±11.15 | 70.47±10.64 | 70.47±10.41 | 72.33±11.16 | 75.22±12.15 | 79.02±12.98 | 78.16±11.92 | 81.57±18.16 | 0.733 |
| ALT, U/L | 16.80±15.48 | 14.18±9.75 | 14.86±13.09 | 17.00±14.42 | 21.47±23.11 | 26.97±26.84 | 28.16±28.96 | 23.00±10.03 | 0.691 |
| AST, U/L | 19.86±9.98 | 18.58±6.38 | 19.01±8.83 | 19.88±9.32 | 22.06±14.39 | 25.03±16.46 | 27.65±20.65 | 22.00±6.48 | 0.034 |
| TP, g/L | 76.21±3.93 | 74.92±4.15 | 75.64±3.85 | 76.40±3.88 | 77.16±4.06 | 77.40±4.26 | 77.80±5.02 | 79.91±5.20 | 0.575 |
| Alb, g/L | 45.52±2.45 | 45.12±2.81 | 45.44±2.44 | 45.58±2.42 | 45.51±2.50 | 45.35±2.77 | 45.43±2.59 | 46.64±2.48 | 0.037 |
| Glo, g/L | 30.69±3.44 | 29.80±3.43 | 30.20±3.30 | 30.82±3.42 | 31.65±3.62 | 32.04±3.84 | 32.37±4.09 | 33.27±3.76 | 0.779 |
| GGT, U/L | 19.96±17.25 | 14.96±8.35 | 16.96±12.21 | 20.46±18.04 | 26.44±23.07 | 32.70±25.04 | 44.31±40.83 | 33.14±11.82 | 0.554 |

eTable 3. Characteristics of female participants with stratified UA level (continued)

| Variables | Overall | Stratified UA group | | | | | | | *P* for trend |
| --- | --- | --- | --- | --- | --- | --- | --- | --- | --- |
|  |  | <150 | 150~249 | 250~349 | 350~449 | 450~549 | 550~649 | ≥650 |  |
| TBIL, μmol/L | 12.28±4.60 | 12.42±4.77 | 12.37±4.57 | 12.27±4.46 | 12.04±5.36 | 11.76±4.26 | 11.39±3.42 | 9.61±3.29 | 0.708 |
| IBIL, μmol/L | 9.03±3.45 | 9.11±3.50 | 9.06±3.32 | 9.04±3.31 | 8.92±4.46 | 8.76±3.23 | 8.38±2.44 | 6.87±2.39 | 0.56 |
| DBIL, μmol/L | 3.24±1.35 | 3.31±1.39 | 3.30±1.42 | 3.24±1.31 | 3.11±1.26 | 3.00±1.17 | 3.01±1.18 | 2.74±0.92 | 0.055 |
| TC, mmol/L | 4.50±0.86 | 4.32±0.72 | 4.39±0.81 | 4.54±0.87 | 4.71±0.94 | 4.93±0.95 | 4.93±1.00 | 4.35±0.42 | 0.813 |
| HDL, mmol/L | 1.44±0.30 | 1.57±0.28 | 1.51±0.29 | 1.43±0.30 | 1.32±0.28 | 1.23±0.26 | 1.17±0.28 | 1.18±0.37 | 0.824 |
| LDL, mmol/L | 2.63±0.73 | 2.38±0.58 | 2.51±0.69 | 2.67±0.73 | 2.83±0.80 | 2.98±0.86 | 2.90±0.94 | 2.18±0.52 | 0.612 |
| TG, mmol/L | 1.12±0.83 | 0.90±0.48 | 0.93±0.52 | 1.16±0.84 | 1.52±1.19 | 1.93±1.38 | 2.23±1.92 | 2.25±1.12 | 0.588 |
| SCr, μmol/L | 60.20±9.60 | 54.59±7.48 | 57.73±7.75 | 60.87±8.62 | 64.29±11.66 | 70.46±24.36 | 79.71±32.57 | 115.86±121.97 | 0.807 |
| eGFR, mL/min/1.73m^2 c^ | 119.13±23.26 | 134.45±27.31 | 125.10±22.49 | 117.18±22.09 | 109.66±24.22 | 100.89±27.65 | 88.65±26.90 | 83.63±34.36 | 0.709 |
| Glu, mmol/L | 5.16±0.87 | 5.15±1.28 | 5.11±0.84 | 5.16±0.83 | 5.32±1.04 | 5.55±1.02 | 5.41±1.15 | 5.10±0.74 | 0.392 |
| UpH | 6.17±0.66 | 6.43±0.68 | 6.27±0.67 | 6.14±0.65 | 6.00±0.63 | 5.89±0.62 | 5.71±0.60 | 5.79±0.39 | 0.335 |

Abbreviations: UA, serum uric acid; BMI, body mass index; CHD, coronary heart disease; SBP, systolic blood pressure , DBP, diastolic blood pressure; ALT, alanine aminotransferase; AST, aspartate aminotransferase; TP, total protein; Alb, albumin; Glo, globulin; GGT, γ-glutamyl transpeptidase; TBIL, total bilirubin; IBIL, indirect bilirubin; DBIL, direct bilirubin; TC, total cholesterol; HDL, high-density lipoprotein cholesterol; LDL, low-density lipoprotein cholesterol; TG, triglycerides; SCr, serum creatinine; eGFR, estimated glomerular filtration rate; Glu, fasting glucose; UpH, Urine pH.

^a^ Calculated as weight in kilograms divided by height in meters squared.

^b^ Classified according to Asian-specific criteria.

^c^ Calculated using the CKD-EPI equation (Details can be found in Methods section).

**eTable 4. Sensitivity analysis for missing data**

|  | Without missing data ^a^ | | | | With missing data ^a^ | | | |
| --- | --- | --- | --- | --- | --- | --- | --- | --- |
|  | OR ^b^ | LCI | UCI | *P* | OR ^b^ | LCI | UCI | *P* |
| Unadjusted | 1.179 | 1.166 | 1.192 | < 0.001 | 1.184 | 1.172 | 1.196 | < 0.001 |
| Model 1 ^c^ | 1.094 | 1.079 | 1.108 | < 0.001 | 1.098 | 1.085 | 1.112 | < 0.001 |
| Model 2 ^c^ | 1.079 | 1.064 | 1.094 | < 0.001 | 1.084 | 1.070 | 1.098 | < 0.001 |
| Model 3 ^c^ | 1.073 | 1.057 | 1.090 | < 0.001 | 1.073 | 1.057 | 1.090 | < 0.001 |
| Mean Difference ^d^ | 31.96 | 29.61 | 34.28 | - | 32.71 | 31.00 | 34.16 | - |
| Nonlinear Trend ^e^ | - | - | - | < 0.001 | - | - | - | < 0.001 |

LCI, lower 95% confidence interval; UCI, upper 95% confidence interval.

^a^ Descriptions for missing data can be found in the Table 1 footnote.

^b^ OR for the Unadjusted model and model 1, 2, 3 is odds ratio.

^c^ Descriptions for models can be found in Methods-Statistical Analyses section.

^d^ Calculated applying bootstrap method. Details can be found in Methods-Statistical Analyses section.

^e^ Calculated applying restricted cubic spline model with 5 knots. Details can be found in Methods-Statistical Analyses section.
